# Effect of Carbonic Anhydrase on CO_2_ Separation Performance of Thin Poly(amidoamine) Dendrimer/Poly(ethylene glycol) Hybrid Membranes

**DOI:** 10.3390/membranes9120167

**Published:** 2019-12-05

**Authors:** Shuhong Duan, Teruhiko Kai, Shin-ichi Nakao

**Affiliations:** Research Institute of Innovative Technology for the Earth (RITE), 9-2 Kizugawadai, Kizugawa-shi, Kyoto 619-0292, Japan; kai.te@rite.or.jp (T.K.); maku@mvg.biglobe.ne.jp (S.-i.N.)

**Keywords:** CO_2_ separation, poly(amidoamine) dendrimer, carbonic anhydrase (CA), rate-limiting step, membrane thickness

## Abstract

The effect of carbonic anhydrase (CA) on the separation performance of thin poly(amidoamine) (PAMAM) dendrimer/poly(ethylene glycol) (PEG) hybrid membranes was investigated. CA, a type of enzyme, was used to promote CO_2_ hydration and dehydration reactions and to assess whether these reactions were the rate-limiting step in CO_2_ permeation through the membrane. The relationship between the membrane thickness and the CO_2_ permeance was evaluated in CO_2_/H_2_ or CO_2_/He separation using PAMAM/PEG hybrid membranes (thickness: 10–100 μm) with and without CA. Without CA, the CO_2_ permeance of PAMAM/PEG hybrid membranes was not inversely proportional to the membrane thickness. On the other hand, with CA, the CO_2_ permeance was inversely proportional to the membrane thickness. It was implied that, without CA, the rate-limiting step of CO_2_ transport was either the CO_2_ hydration reaction at the feed side or the CO_2_ dehydration reaction at the permeate side. On the other hand, with CA addition, the rate-limiting step of CO_2_ transport was diffusion, and CO_2_ permeance could be increased without sacrificing the selectivity by reducing membrane thickness. The effect of the position of CA (i.e., on the surface and/or reverse surface) on CO_2_ separation performance was investigated to evaluate which reaction was the rate-limiting step of CO_2_ permeation through the membrane. It was suggested that the rate-limiting step of CO_2_ permeation was CO_2_ dehydration reaction at the permeate side.

## 1. Introduction

CO_2_ capture and storage (CCS) is widely accepted as an important option for mitigating climate change, and has been attracting worldwide attention [1]. CO_2_ capture includes CO_2_ separation from flue gas (post-combustion, CO_2_/N_2_), CO_2_ separation from natural gas (CO_2_/CH_4_), and CO_2_ separation from integrated gasification combined cycle (IGCC) processes (pre-combustion, CO_2_/H_2_) [2,3]. For practical application of the CCS technology, cost-effective methods for CO_2_ capture are required. Many studies have focused on the development of effective CO_2_ capture and separation technologies. Membrane separation would be one of the most promising approaches among them in terms of technological and economic perspectives. Polymeric membranes [4,5], inorganic membranes [6,7], ionic liquid membranes [8], and facilitated transport membranes [9,10] were studied for CO_2_ separation. Most of the CO_2_ selective membranes were developed for post-combustion. If CO_2_ selective membranes are used for pre-combustion (high pressure gas), H_2_ can be kept at high pressure in the retentate side and directly fed to gas turbine without compression, and CO_2_ can transport though the membrane without using vacuum pump in the permeate side, so it is energy and cost saving to apply a CO_2_ selective membrane for pre-combustion. However, it is very difficult to separate CO_2_ over H_2_, because the molecular size of CO_2_ is greater than that of H_2_, and only limited numbers of CO_2_ selective membranes were reported for this purpose [11,12,13]. 

It was reported that poly(amidoamine) (PAMAM) dendrimer showed excellent CO_2_/N_2_ separation performance in a liquid immobilized membrane (ILM) under 200 kPa low pressure [14]. In our group, PAMAM dendrimer/crosslinked-polymer hybrid membranes, such as PAMAM dendrimer/poly(ethylene glycol) (PEG) hybrid membranes [11,12,13] have been developed by immobilizing PAMAM dendrimer into the cross-linked polymer matrix for use in high pressure CO_2_ separation. We found that these membranes show high CO_2_/H_2_ separation performance at pressurized conditions, and have the potential to be applied for pre-combustion. In our previous paper, we developed hybrid membranes composed of PAMAM dendrimer and polyethylene glycol dimethacrylate (PEGDMA) and a compatible cross-linker 4GMAP that enabled thickness less than 100 µm [13]. However, we found that CO_2_ permeance (Q_CO₂_) was not inversely proportional to thickness, and CO_2_/H_2_ separation performance was reduced from ca. 30 µm to ca. 10 µm by reducing the membrane thickness. The experimental results were explained by the facilitated transport theory [15]. The permeances of both CO_2_ and H_2_ increased with decreasing thickness. However, since the membrane thickness dependence of Q_CO₂_ was lower than that of H_2_, selectivity of CO_2_ over H_2_ decreased with decreasing the membrane thickness. 

**Figure 1:**
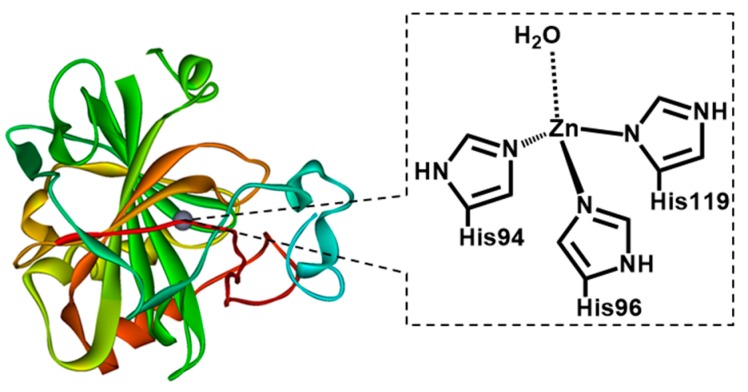
Carbonic anhydrase active region.

**Figure 2:**
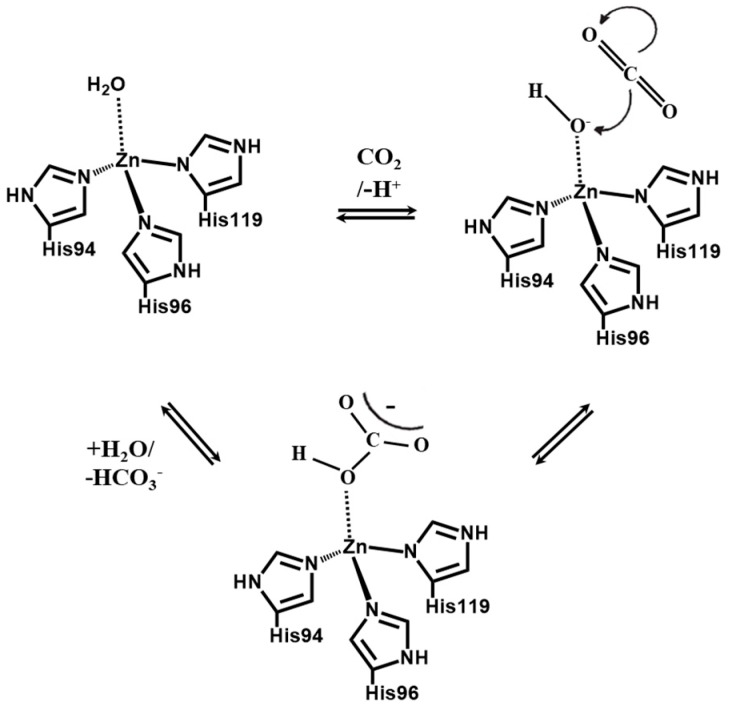
CO_2_ hydration mechanism of carbonic anhydrase.

In this paper, we investigated the effect of enzyme on the CO_2_ separation performance, in order to find the solution to obtain both high Q_CO₂_ and selectivity for the thin PAMAM/PEG hybrid membranes. Carbonic anhydrase (CA) is a well-known enzyme to promote CO_2_ hydration and dehydration reaction (CO_2_ + H_2_O ⇔ H_2_CO_3_) [16,17]. CA is an efficient catalyst for CO_2_ hydration and dehydration with a turn over number of 106 mol-CO_2_/(mol-CA s) at the maximum. The carbonic anhydrase active region is shown in Figure 1 [16]. The CO_2_ hydration mechanism of carbonic anhydrase is shown in Figure 2 [16,17]. A carbon dioxide molecule is attached to the Zn^2+^ active site to form a meta-stable complex. The complex is then attacked by a Lewis base (OH^−^) to produce bicarbonates (HCO_3_^−^). In this two-step process, CO_2_ is converted to HCO_3_^−^ and the active site in the CA is left un-reacted. Therefore, in this paper, in order to overcome the limitation of the CO_2_ separation properties, the effect of CA on separation properties was investigated. As far as the authors know, this paper is the first to report the effects of CA on CO_2_ separation performance of the facilitated transport membranes in detail, such as the relationship between Q_CO₂_ and membrane thickness, rate-limiting step of CO_2_ permeation, etc. 

## 2. Materials and Methods 

### 2.1. Materials

PEGDMA (average Mn 750), 1-hydroxycyclohexyl phenylketone, PAMAM dendrimer in methanol (0th generation, 50 wt.%), and carbonic anhydrase (CA) from bovine erythrocytes, were obtained from Sigma-Aldrich (MO, USA). Other organic and inorganic materials were reagent grade and used without further purification. Polyethersulfone (PES) porous support membrane with 30 kDa NMWCO was purchased from Millipore Com (Tokyo, Japan). A compatible cross-linker, 4GMAP, was synthesized by the reaction between PAMAM dendrimer (G0) and glycidyl methacrylate, as shown in [13].

### 2.2. Membrane Preparation

A polymeric membrane was fabricated by photopolymerization of PEGDMA in the presence of PAMAM dendrimer in water. The composition of precursor solution is PAMAM (50 wt %), PEGDMA (42.5 wt %) and 4GMAP (7.5 wt %) in water.

**Figure 3:**
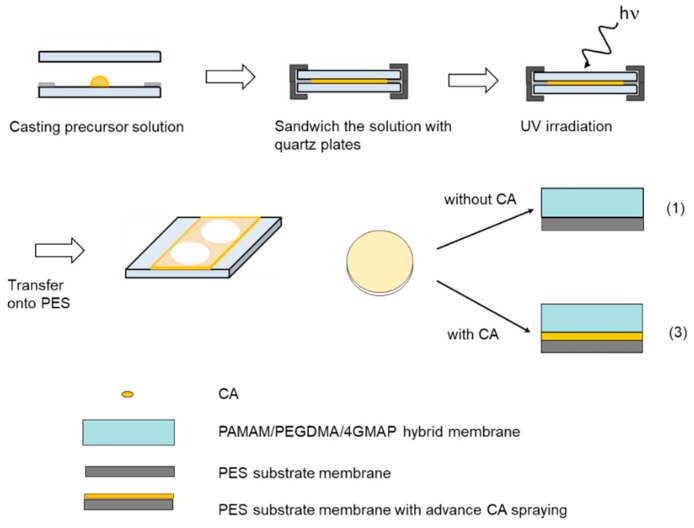
Membrane fabrication of thin poly(amidoamine) (PAMAM) dendrimer/poly(ethylene glycol) (PEG) hybrid membranes with or without carbonic anhydrase (CA).

A schematic diagram of membrane fabrication of thin PAMAM/PEGDMA/4GMAP with or without CA hybrid membranes is shown in Figure 3. The hybrid membrane was prepared by casting precursor solution on a quartz plate, followed by the UV curing at 312 nm UV for 1.5 min and transferred onto PES support membrane. The membrane thickness was controlled by sandwiching the reaction mixture between quartz plates with stainless steel spacers (10–100 µm in thick). 

**Figure 4:**
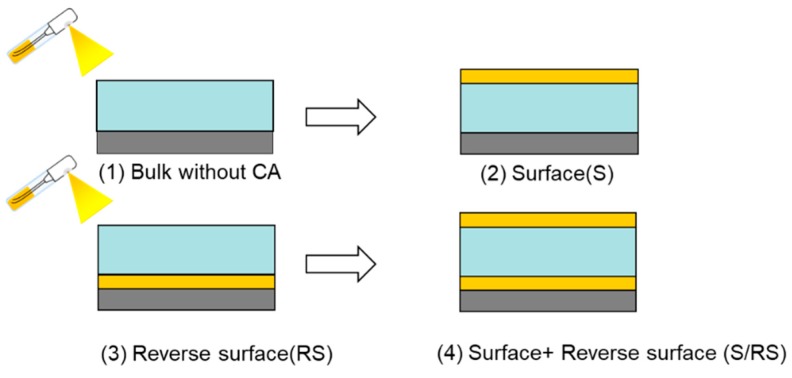
CA addition by a spray method CA 0.5 wt % aqueous solution onto the membranes with 20 μm gap.

To investigate the effect of CA additive, composite membranes with the selectivity layer PAMAM/PEGDMA/4GMAP shown were prepared with the reverse surface, with CA coated on the surface of the PES support membrane by spraying in advance (Figure 3 (3)). A schematic diagram of CA addition by spray method of CA onto the membranes is shown in Figure 4. The membrane thickness is determined by a KeyenceVHX-1000 digital microscope (Tokyo, Japan) [14].

### 2.3. Gas Separation Experimental

A schematic diagram of the gas separation experiment setup is shown in [18]. A CO_2_/H_2_ or CO_2_/He (80/20 by vol.) gas mixture was humidified at 80% relative humidity and then fed to a flat-sheet membrane cell at a flow rate of 100 mL/min. As we reported in our previous papers, our membrane needs relative humidity as high as 80% RH to show high separation performance [18]. The CO_2_ partial pressures of the feed side was 560 kPa (total feed pressure 700 kPa). Dry Ar was supplied at a flow rate of 10 mL/min to the permeate side of the cell as a sweep gas. The test operating temperatures were at 40 °C. The CO_2_ and He concentrations in both feed and permeate gas were measured by gas chromatography. Permeance, Q, and selectivity, CO_2_/H_2_ or CO_2_/He were calculated as expressed in [18]. A CO_2_/H_2_ (80/20 by volume) gas mixture was used for the gas separation experimental of Section 3.1. A CO_2_/He (80/20 by volume) gas mixture was used for the gas separation experimental of Section 3.2 and Section 3.3. He was used instead of H_2_ for the safety reason. The relationship between Q_He_ (permeance of He) and Q_H₂_ (permeance of H_2_) was as follows: Q_He_ ≈ 0.8 Q_H₂_. On the other hand, Q_CO₂_ was almost the same for both CO_2_/He and CO_2_/H_2_ separation. 

## 3. Results and Discussion

### 3.1. Effect of Membrane Thickness on CO_2_ Permeance and CO_2_/H_2_ Selectivity

**Figure 5:**
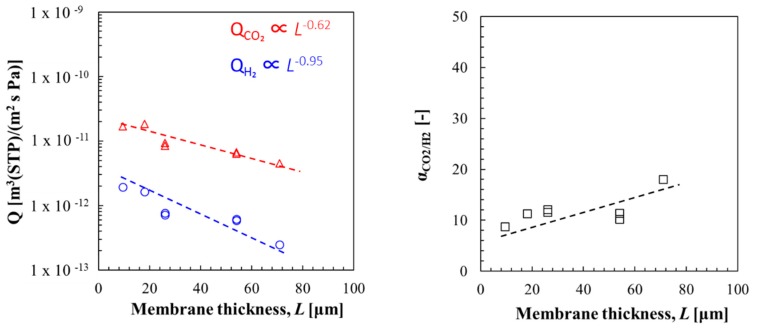
Effect of membrane thickness (*L*) on CO_2_ permeance and CO_2_/H_2_ selectivity.

The effect of membrane thickness on CO_2_ permeance and CO_2_/H_2_ selectivity was studied at 560 kPa of CO_2_ partial pressure and 80% RH at 40 °C with hybrid membranes of PAMAM/PEGDMA/4GMAP = 50/42.5/7.5 by wt %; thickness = 10–100 µm. It was found that thinner membranes gave higher CO_2_ permeation properties, as shown in Figure 5. However, CO_2_ permeance was not inversely proportional to membrane thickness (Q_CO₂_ ∝ *L*^−0.62^). On the other hand, H_2_ permeance was inversely proportional to membrane thickness (Q_H₂_ ∝ *L*^−0.95^). As a result, selectivity of CO_2_ over H_2_ decreased as the membrane thickness decreased.

The amino group contributes to transport of CO_2_ though membrane as a bicarbonate ion (HCO_3_^−^) in the wet membrane, while H_2_ is only transported by a solution–diffusion mechanism [16]. The rate-limiting step of CO_2_ permeation was the reaction from CO_2_ to a bicarbonate ion (HCO_3_^−^) at the feed side, or the reaction from HCO_3_^−^ to CO_2_ at the permeate side. Since the membrane thickness dependence of Q_CO₂_ is lower than that of H_2_, selectivity of CO_2_ over H_2_ decreased as the membrane thickness decreased. The experimental results were explained by the facilitated transport theory [15,16]. To obtain high Q_CO₂_ and selectivity, CA was added into CO_2_ carrier (PAMAM) to obtain much higher reactivity with CO_2_ in next section.

### 3.2. Effect of CA Addition on the CO_2_ Separation Properties 

**Figure 6:**
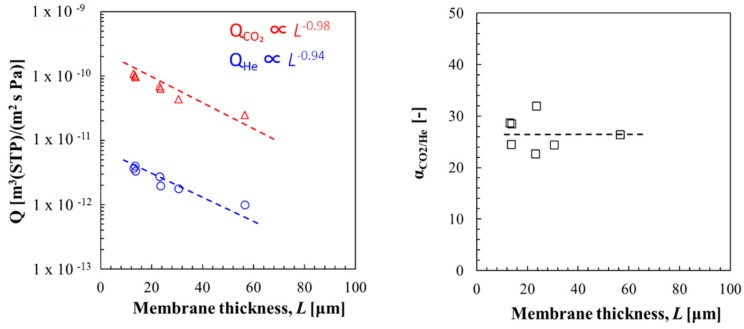
Effect of CA addition on the CO_2_ separation properties of PAMAM/PEG hybrid membranes.

The effect of CA addition and membrane thickness on CO_2_ permeance and CO_2_/H_2_ selectivity was studied at 560 kPa of CO_2_ partial pressure and 80% RH at 40 °C with hybrid membranes of PAMAM/PEGDMA/4GMAP = 50/42.5/7.5 by weight ratio with addition 1 wt % CA (membrane thickness = 10–60 µm). As can be seen from Figure 6, CO_2_ permeance was inversely proportional to membrane thickness (Q_CO₂_ ∝ *L*^−0.98^). He permeance was also inversely proportional to membrane thickness (Q_He_ ∝ *L*^−0.94^). As a result, CO_2_/He selectivity kept constant. It was suggested that CA addition enhanced the reaction rate of CO_2_ hydration at the feed side and the dehydration at the permeate side, and that the rate-limiting step of CO_2_ transport rate became diffusion.

CO_2_ permeace of as high as 2.47 × 10^−11^ m^3^ (STP)/(m^2^ s Pa) accompanied with a CO_2_/He selectivity of 26.8 was achieved by the membrane with CA addition, ca. 55 μm thick. The enhancement in CO_2_ permeace is 270% compared with the membrane without CA addition (Q_CO₂_: 6.71 × 10^−12^ m^3^ (STP)/(m^2^ s Pa), ca. 55 μm thick). CO_2_ permeace of as high as 1.08 × 10^−10^ m^3^ (STP)/(m^2^ s Pa) accompanied with a CO_2_/He selectivity of 28.7 was achieved by the membrane with CA addition, ca. 15 μm thick. The enhancements in CO_2_ permeace is 490% compared with the membrane without CA addition (Q_CO₂_: 1.84 × 10^−11^ m^3^ (STP)/(m^2^ s Pa), ca. 15 μm thick). It was indicated that CA addition was effective to break though the limitation of CO_2_ permeation of the thin facilitated transport membrane. 

### 3.3. Effect of Position of CA on CO_2_ Separation Performance 

**Figure 7:**
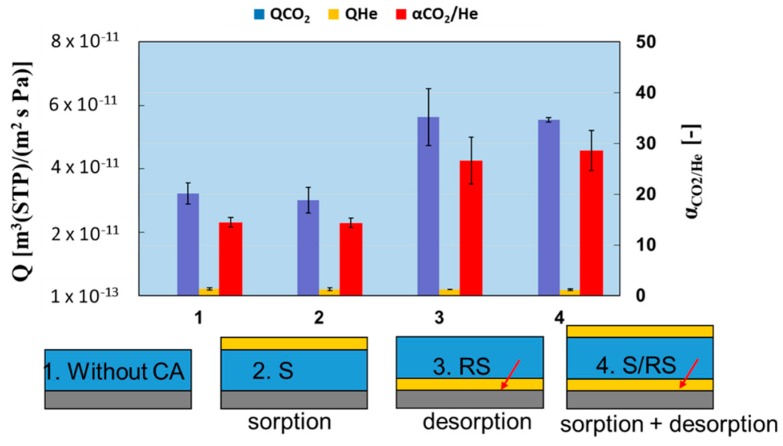
Effect of CA position on the CO_2_ separation properties.

The effect of position of CA on CO_2_ separation performance was studied at 560kPa of CO_2_ partial pressure and 80% RH at 40 °C, using hybrid composite membranes of PAMAM/PEGDMA/4GMAP = 50/42.5/7.5 (weight %) with CA at different positions: (1) without CA; (2) S: surface; (3) RS: reverse surface; (4) S/RS. The thicknesses of these membranes were ca. 20 µm. The results are shown in Figure 7. CO_2_ permeance of the membrane with CA at S position was not higher than that of membrane without CA. On the other hand, the membranes with CA at RS and S/RS positions showed CO_2_ permeance and selectivity around twice as high as that of the membrane without CA or the membrane with CA at S position. From these results, it could be seen that the existence of CA at the RS position was more important than the S position. RS position is the place where CO_2_ dehydration occurs at the permeate side, and the S position is the place where CO_2_ hydration occurs at the feed side. Therefore, it was suggested that the rate-limiting step of CO_2_ permeation was CO_2_ dehydration reaction at the permeate side. These findings will be useful in the development of the facilitated transport membrane with high CO_2_ separation properties. Detailed research on the CO_2_ permeation mechanism is ongoing.

## 4. Conclusions

Effect of CA enzyme on CO_2_ separation performance of thin poly(amidoamine) dendrimer/poly(ethylene glycol) hybrid membranes was investigated by examining the relationship between membrane thickness and CO_2_ permeance using the membranes with or without CA. CO_2_ permeance was inversely proportional to membrane thickness with CA, and not proportional to membrane thickness without CA. The addition of CA exhibited significantly enhanced CO_2_ separation performances. The membrane with CA at the permeate side showed higher CO_2_ separation performance than that of the membrane with CA at the feed side. Therefore, it was indicated that the rate-limiting step of CO_2_ permeation was CO_2_ dehydration reaction at the permeate side. These findings will be useful in the development of the CO_2_ facilitated transport membrane with high CO_2_ separation properties.
